# Insights from Computational Dynamic Active Site Mapping into Substrate Recognition and Mutation-Induced Dysfunction in Human Tyrosinase

**DOI:** 10.3390/ijms27041937

**Published:** 2026-02-18

**Authors:** Monika B. Dolinska, Yuri V. Sergeev

**Affiliations:** National Eye Institute, National Institutes of Health, Bethesda, MD 20892, USA

**Keywords:** tyrosinase, oculocutaneous albinism type 1 (OCA1), OCA1-related genetic mutations, molecular dynamic simulations, molecular docking, multi-substrate recognition

## Abstract

The ability of enzymes to recognize and process structurally diverse substrates is fundamental to metabolic flexibility and biological regulation. In melanin biosynthesis, human tyrosinase (Tyr) catalyzes the oxidation of several chemically distinct intermediates, including L-tyrosine, L-DOPA, DHICA, and DHI. Although its catalytic chemistry is well established, the structural basis of substrate selectivity and how it is altered by disease-associated mutations remains unclear. Using molecular docking and molecular dynamics simulations, we mapped the Tyr active site and identified 23 evolutionarily conserved residues that mediate multi-substrate recognition and binding. Across all substrates, binding induces coordinated conformational responses, particularly within an anchoring region (334–347) that provides electrostatic and hydrophobic steering, and a flexible gating loop (374–386) that modulates access and stabilizes bound intermediates. The OCA1B-associated P406L mutation, although distant from the catalytic core, disrupts long-range dynamic coupling and impairs loop flexibility, while 25 ClinVar-listed genetic variants at substrate-interacting residues weaken active-site organization, underscoring the sensitivity of Tyr’s dynamic network to perturbation. Integrating these findings, we propose an ordered multi-substrate binding mechanism in which substrates are first guided by the anchoring region, then aligned by the universal triad, and finally refined through loop-mediated, substrate-specific contacts. Our work suggests a dynamic framework that could be useful for understanding human tyrosinase catalysis, genetic mutation impact, and future engineering strategies.

## 1. Introduction

Human tyrosinase (Tyr) is a copper-dependent, transmembrane glycoenzyme that catalyzes the initial and rate-limiting steps of melanin biosynthesis: the hydroxylation of L-tyrosine to L-DOPA and the subsequent oxidation of L-DOPA to dopaquinone [1,2]. These early reactions direct the pathway toward eumelanin or pheomelanin production, depending on redox conditions and the activity of additional enzymes such as tyrosinase-related proteins 1 (Tyrp1) and 2 (Tyrp2), and the availability of cysteine [3]. Melanin, in turn, is the primary pigment responsible for human skin, hair, and eye color and plays a crucial protective role against ultraviolet (UV) radiation and oxidative stress [4,5].

Although the general catalytic mechanism of Tyr is well established, the structural basis for its ability to accommodate chemically diverse substrates remains poorly understood [1,2]. Tyr interacts with ligands that differ in polarity, hydrophobicity, aromaticity, conformational flexibility, charge distribution, and reactivity, from L-tyrosine and L-DOPA to downstream intermediates such as 5,6-dihydroxyindole-2-carboxylic acid (DHICA) and 5,6-dihydroxyindole (DHI). While L-tyrosine initiates melanin synthesis and L-DOPA serves both as a product and a substrate, DHICA and DHI are more oxidized intermediates; Tyrp1 preferentially processes DHICA [6,7], whereas DHI can undergo spontaneous or enzymatic oxidation [8]. These physicochemical differences suggest that Tyr must undergo substrate-specific conformational changes to enable effective catalysis and maintain substrate selectivity.

Tracking the dynamic behavior of the enzyme and its substrates throughout the binding process remains a significant challenge in the experimental system. As a result, how human Tyr structurally responds to different substrates and how disease-causing mutations disrupt this response remain poorly understood. This gap has clinical relevance. Mutations in the TYR gene cause oculocutaneous albinism type 1 (OCA1), a genetic condition characterized by absent (OCA1A) or reduced (OCA1B) melanin production in the skin, hair, and eyes that significantly increases the risk of skin cancer and leads to vision problems [9]. Conversely, excessive melanin production or abnormal activation of melanocytes can lead to hyperpigmentation disorders such as melasma or melanoma [10,11]. As such, Tyr has emerged as a key pharmacological target: inhibitors are employed to suppress hyperpigmentation [12], while strategies to activate or stabilize Tyr are under investigation for hypopigmentation disorders [13,14]. Despite its clinical importance, most Tyr-targeting compounds have been identified empirically and often suffer from low specificity or off-target toxicity, underscoring the need for rational structure-based drug design. A detailed understanding of native substrate binding could aid the development of substrate analogs, allosteric modulators, or chemical chaperones.

Substrate binding frequently induces conformational changes that regulate enzymatic function through loop dynamics and active-site remodeling. These motions are essential for catalysis, as seen in diverse systems where substrate access, metal repositioning, or product release is mediated by structural flexibility [15,16]. Loop mobility also underlies the evolution of new enzyme activities by enabling functional adaptation [17]. While these mechanisms are well-characterized in other enzymes, such dynamics remain unexplored in human Tyr across its physiological substrates.

Previously, we demonstrated in silico that the human intra-melanosomal domain of Tyr can accommodate L-tyrosine, L-DOPA, DHICA, and DHI within the same active site through conserved residue and copper coordination [18]. Despite their differences, all four substrates formed stable complexes with Tyr, supporting its catalytic versatility. The P406L variant showed reduced capacity for stable substrate interaction, in line with impaired enzymatic performance. Longer molecular dynamics (MD) simulations revealed that P406L increases flexibility within the catalytic domain under unfolding conditions, promoting a molten globule-like state indicative of structural and functional destabilization [19]. These findings were supported by in vitro studies demonstrating decreased enzymatic activity and stability of P406L relative to the wild type [20], with kinetic analyses revealing altered turnover rates and thermodynamic signatures [21].

In this study, we extend these observations by comparing all four substrate-specific conformational changes across 23 interacting residues located in the catalytic site surface area that consistently mediate recognition and binding in wild-type Tyr and its disease-associated mutants. Within this interaction set, we identify substrate-specific residues that uniquely contact L-tyrosine, L-DOPA, DHICA, and DHI and reflect each ligand’s chemistry and functional role in melanogenesis, and a small group of universal residues engaged by all substrates, providing a shared structural framework for anchoring and aligning chemically diverse ligands. By integrating these residue-level interactions with all-atom MD simulations of the human intra-melanosomal domain of Tyr bound to four melanogenic intermediates, we characterize how substrate engagement may modulate residue flexibility, atomic deviation, solvent exposure, secondary-structure transitions, contact networks, and dynamic cross-correlation patterns. These analyses reveal both conserved and ligand-dependent structural adaptations and form the basis of a proposed ordered binding mechanism, in which substrates are first guided by an electrostatic/hydrophobic anchoring region, then aligned by a universal triad, and finally refined through loop-mediated substrate-specific contacts. This computational framework provides a mechanistic view of how Tyr might achieve both broad substrate selectivity and catalytic precision, features that remain difficult to resolve through experimental approaches alone.

We further examined the structural consequences of the OCA1B-associated P406L mutation. Although spatially distal from the catalytic center, P406L does not disrupt catalysis directly but instead induces long-range allosteric perturbations that destabilize peripheral loops and weaken active-site coordination. This is consistent with emerging evidence that distal mutations impair enzyme function by altering structural communication networks [22]. Analysis of an additional 25 ClinVar-listed genetic variants at substrate-interacting residues similarly shows that many destabilize active-site architecture and disrupt dynamic coupling [23].

Together, these results reveal how substrate-specific contacts, universal anchoring residues, a loop-mediated refinement step, and distal pathogenic mutations may collectively shape the dynamic architecture of Tyr. These findings further support an ordered multi-substrate binding mechanism in which distinct structural elements may act sequentially to guide, align, and refine ligand interactions. This integrated view of substrate engagement and mutational impact provides a generalizable model for allosteric regulation in enzymes and offers mechanistic insight relevant to variant interpretation, protein engineering, and structure-guided design targeting Tyr and related copper oxidases.

## 2. Results

### 2.1. Selection of Key Residues Involved in Tyr Substrate Binding to Map Its Active Site

In the absence of an experimentally determined structure of human Tyr, a homology model of its intra-melanosomal domain was generated using human Tyrp1 (PDB ID: 5M8L) as the template. Tyrp1 was chosen due to its close evolutionary relationship to Tyr and strong conservation within the catalytic core and dicopper active site. Sequence alignment showed ~41% overall identity between Tyr and Tyrp1, increasing to ~43% within the catalytic region (residues 180–390) and to ~63% among the substrate-interacting residues analyzed here. All copper-coordinating histidines are conserved, supporting the appropriateness of this model for comparative analysis of substrate binding and active-site dynamics while avoiding overinterpretation beyond the conserved catalytic region.

**Figure 1:**
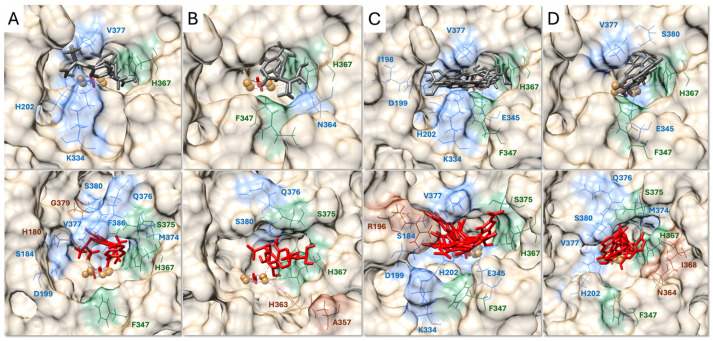
Surface map of key residues involved in small-molecule substrates binding to Tyr. Computational docking of L-tyrosine (**A**), L-DOPA (**B**), DHICA (**C**), and DHI (**D**) into the Tyr active site. Ligands are shown as gray sticks in the initial docked pose (top panels) and as red sticks after 20 ns of MD simulation (bottom panels). Interacting residues are depicted as wires: substrate-specific residues in brown, universal residues engaged by all substrates in green, and the remaining interacting residues in blue. The Tyr surface is rendered in light tan with local surface coloring corresponding to the residue color scheme. Copper atoms (CuA and CuB) are shown as orange spheres, and the bridging O_2_ molecule is shown in red.

To investigate Tyr’s substrate-binding modes, we performed molecular docking of L-tyrosine, L-DOPA, DHICA, and DHI (Figure 1, Table S1).

For all cases in which a ligand remained bound to Tyr during MD simulations, results were averaged across replicates to obtain consistent measures of structural and dynamic changes. These analyses reflect short-timescale (20 ns), active-site-level dynamics and are intended to compare relative ligand binding stability and interaction patterns, rather than to capture slow conformational transitions or large-scale domain motion. Representative frames taken every 1 ns over a 20 ns interval were analyzed to identify residues involved in substrate recognition and stabilization. We focused on hydrogen bonding, hydrophobic contacts, and π–π stacking. In addition, we included the Cu-coordinating histidines (H211 and H390), which, although not directly participating in substrate binding, are functionally relevant to the catalytic site architecture.

**Figure 2:**
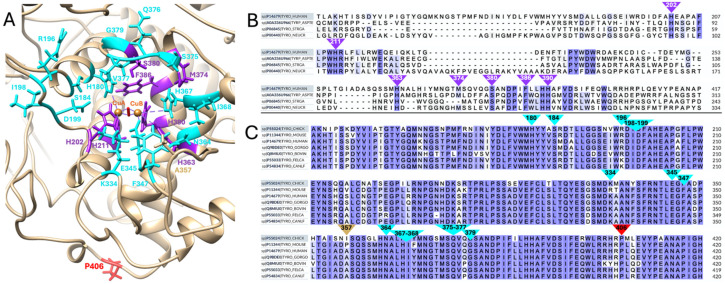
Sequence and structural conservation of Tyr across species. Panel (**A**): Homology model of the Tyr intra-melanosomal domain, showing conserved ligand-interacting residues across distant species (purple) and vertebrates (cyan). Residue, A357, is shown in tan. The P406L mutation site is shown in red. Copper atoms in the active site (CuA and CuB), depicted as orange spheres, are stabilized by an O_2_ molecule positioned between them and represented as a red rod. The protein backbone is shown as a tan ribbon. Panel (**B**): Multiple sequence alignment of Tyr proteins from distant species, including human, fungi (Aspergillus, Neurospora), and bacteria (Streptomyces). Conserved residues are highlighted in purple. Tyr ligand-interacting residues are marked with purple triangles. Panel (**C**): Multiple sequence alignment of Tyr proteins from vertebrates (chicken, mouse, human, gorilla, bovine, cat, and dog). Conserved residues are highlighted in purple. Conserved ligand-interacting residues are indicated with cyan triangles, residue A357, conserved due to its hydrophobic property is indicated with brown triangle, and the P406L mutation site is marked with a red triangle. Protein sequences from different species were retrieved and aligned using the UniProtKB database (https://www.uniprot.org/align (accessed on 10 June 2025)).

From these simulations, we identified 23 residues, H180, S184, R196, I198, D199, H202, H211, K334, E345, F347, A357, H363, N364, H367, I368, M374, S375, Q376, V377, G379, S380, F386, and H390, that participate in substrate engagement to varying degrees over the MD trajectory (Figure 1, Table S1). Except for A357, which is conserved primarily due to its hydrophobic character, all residues are conserved either among vertebrates or across more distantly related species, underscoring their evolutionary and functional importance (Figure 2). These species were selected to represent evolutionarily distant but catalytically characterized tyrosinases, spanning vertebrate, fungal, and bacterial enzymes. This comparison was intended to identify conserved features within the catalytic core and active site environment, rather than to imply functional equivalence or universality across all tyrosinase family members.

### 2.2. Substrate-Specific and Universal Interactions

Within this 23-residue interaction set, several positions were uniquely engaged by only one substrate (Figure 1, Table S1). L-tyrosine specifically interacted with H180 and G379, L-DOPA uniquely engaged A357 and H363, DHICA selectively recruited R196, and DHI exclusively contacted I368 and N364. These ligand-specific residues occupy distinct structural regions, including CuA-adjacent histidines, CuB-side coordination elements, and segments of the 374–386 gating loop, indicating that each substrate accesses a characteristic subset of the active site.

In addition to these selective contacts, three residues, H367, F347, and S375, were engaged by all four substrates, forming a conserved set of universal recognition elements. These positions span the CuB-proximal anchoring region and the flexible gating loop, providing shared structural points used by all ligands regardless of their chemical differences. Together, the presence of both substrate-specific and universal contacts demonstrates that Tyr combines a conserved anchoring core with ligand-tailored interactions to bind chemically diverse substrates.

### 2.3. Substrate-Induced Structural and Dynamic Changes

**Figure 3:**
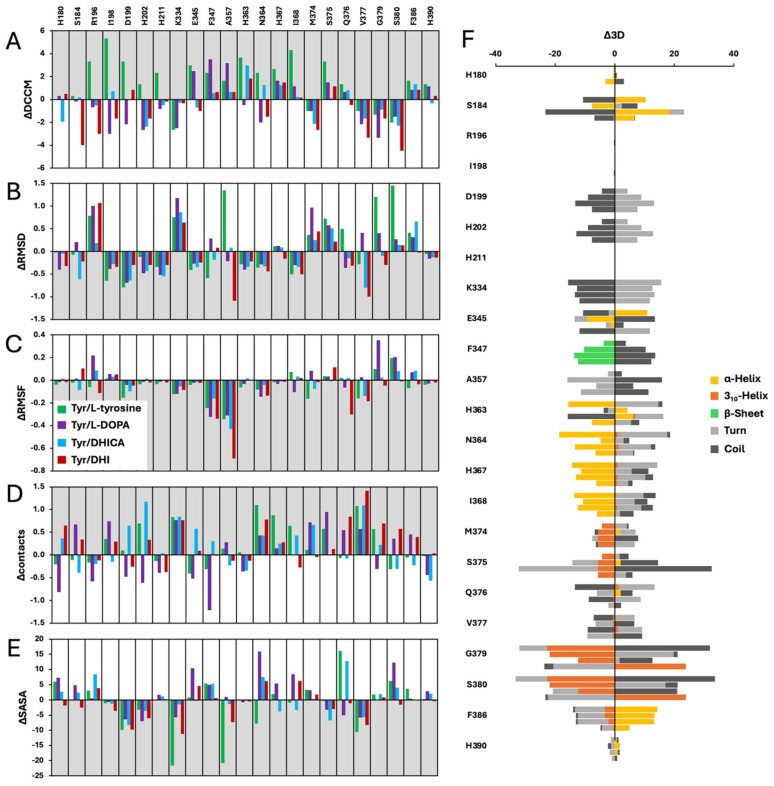
Structural and dynamic effects of substrate binding on Tyr ligand-interacting residues. ΔDCCM (**A**), ΔRMSD (**B**), ΔRMSF (**C**), Δcontacts (**D**), and ΔSASA (**E**) of ligand-interacting residues for Tyr in complex with L-tyrosine (green bars), L-DOPA (purple bars), DHICA (blue bars), and DHI (red bars). The gray background indicates stabilization of the residues in each analysis. (**F**): Per residue binding-induced changes in secondary structure content for Tyr ligand-interacting residues in complexes with L-tyrosine (first bars), L-DOPA (second bars), DHICA (third bars), and DHI (fourth bars). Bars represent secondary structure content, color-coded as follows: α-helix (yellow), 3_10_-helix (orange), β-sheet (green), turn (light gray), and coil (dark gray).

To evaluate how substrate binding affects Tyr dynamics, all 23 interacting residues were assessed across multiple structural and dynamic parameters: dynamic cross-correlation matrices (DCCMs), root mean square deviation (RMSD), root mean square fluctuation (RMSF), per residue contact numbers, solvent-accessible surface area (SASA), and 3D conformational changes (Figure 3).

For each substrate, values were averaged across all binding events, and binding-induced changes were quantified. Even when not statistically significant, the trends were highly consistent: substrate binding triggers distinct structural and dynamic reorganizations within the catalytic core, promoting optimal substrate positioning and catalytic efficiency.

L-tyrosine binding induced a dual effect on Tyr, stabilizing the catalytic core while remodeling surrounding loops (Figure 3, Figures S1 and S2). Residues directly involved in catalysis and structural support (D199, H202, H211, E345, F347, H363, N364, H367, I368, H390) showed increased correlated movements along with reduced RMSD and/or RMSF, and in some cases, an increased number of interatomic contacts, features consistent with a more compact and coordinated catalytic environment. E345 and F386 also gained a helical secondary structure, suggesting local structural stabilization. A357 exhibited increased DCCM and additional contacts accompanied by higher RMSD but reduced RMSF and SASA, suggesting repositioning into a more compact and dynamically coupled conformation. In contrast, channel shaping and loop residues (K334, M374, S375, Q376, G379, S380, F386) showed increased RMSD, often accompanied by decreased DCCM, reduced number of contacts, and elevated SASA or loss of helical structure in favor of coil/turn conformations, features consistent with flexibility during substrate accommodation.

L-DOPA binding selectively stabilized Tyr’s catalytic core while inducing remodeling of loop regions (Figure 3, Figures S1 and S2). Stabilized residues A357, I368, Q376, and H390 displayed increased DCCM, decreased RMSD/RMSF, and, in several cases, additional contacts or helix formation, consistent with a more compact catalytic configuration. Residues D199, H202, H211, and H363 also stabilized but displayed reduced DCCM, indicating a reorganization of correlation networks upon ligand engagement. Loop and channel residues (K334, M374, S375, V377, G379, S380, F386) underwent significant remodeling, characterized by increased RMSD, SASA fluctuations, contact rewiring, and transitions between helix, turn, and coil states. F347 exhibited increased DCCM together with higher RMSD and SASA, reduced RMSF and contacts, and loss of ß-sheet structure, features consistent with a gating transition at this position.

DHICA binding produced selective stabilization of core residues coupled with substantial loop remodeling (Figure 3, Figures S1 and S2). Stabilized residues (D199, H202, E345, F347, V377) showed decreased RMSD/RMSF with an increased number of contacts and reduced SASA, indicating localized stabilization of the catalytic core despite partial loss of DCCM correlations. At the same time, I198, H363, N364, H367, Q376, and F386 exhibited increased DCCM, suggesting recoupling into a new ligand-centered correlation network, with some of these residues also gaining helical content. In contrast, flexible channel residues including K334, M374, S375, and S380 underwent pronounced remodeling, with elevated RMSD and in some cases contact rewiring, SASA increases, and secondary structure transitions from helix to coil or turn, consistent with rearrangement of the channel entrance.

DHI binding stabilized the catalytic core while inducing remodeling of channel loops (Figure 3, Figures S1 and S2). Residues including D199, F347, A357, H363, H367, and H390 showed increased DCCM with decreased RMSD/RMSF, indicating tighter, coordinated dynamics, while H202 stabilized through reduced RMSD/RMSF and added contacts despite reduced correlations. Several residues (H180, E345, N364, Q376, V377, G379, S380, F386) gained contacts or reduced SASA, reflecting strengthened packing. In contrast, loop residues K334, M374, S375, and S380 exhibited increased RMSD, SASA shifts, contact rewiring, and helix/coil transitions, consistent with flexible gating and substrate accommodation.

### 2.4. CuA and CuB Contributions to Substrate Binding

Mapping interacting residues onto the copper centers revealed distinct but coordinated contributions of CuA and CuB. Across all four substrates, CuB and its surrounding loop (374–386) formed the primary recognition interface: H367 contacted every ligand, and CuB-adjacent residues (N364, E345, F347, V377, S375, S380) defined a dynamic gating region that opens and reshapes to accommodate substrates. In contrast, CuA contributes in a substrate-dependent manner. L-tyrosine perturbs H180 and stabilizes H202, whereas DHICA and DHI engage H202 directly, consistent with deeper insertion of more oxidized intermediates toward CuA. Thus, CuB and its flexible loop act as a universal entry and anchoring platform, while CuA provides deeper, substrate-selective tuning of orientation and redox alignment.

### 2.5. Effect of the P406L Mutation on Active Site Dynamics

**Figure 4:**
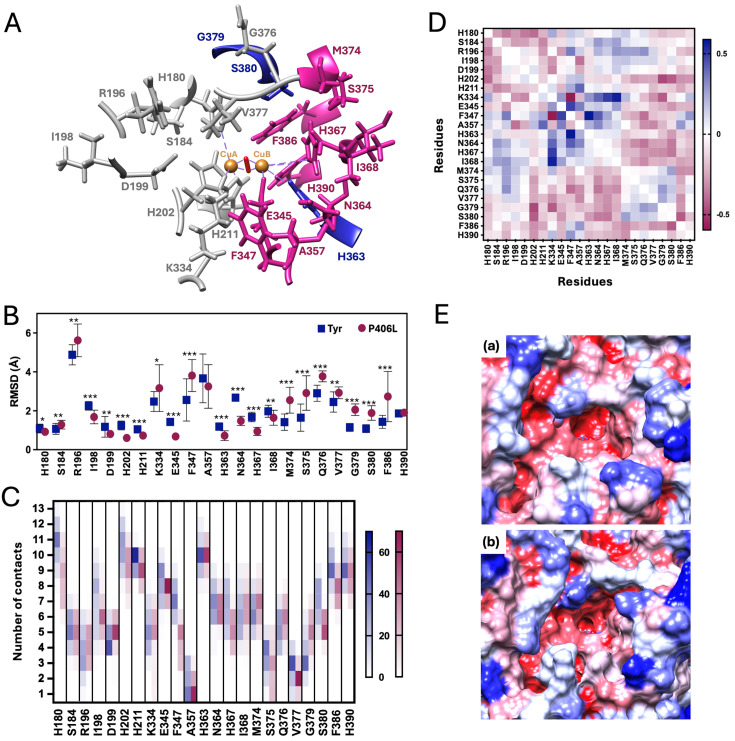
Active site destabilization in the P406L mutant variant. Panel (**A**): Tyr active site showing residues that interact with the Tyr ligands, with predicted per residue secondary structure changes upon the P406L mutation. Residues that lose helicity are colored pink, those that gain helicity are colored blue, and unchanged residues are shown in gray. Copper atoms are depicted as orange spheres, and the bridging O_2_ molecule is shown in red. Panel (**B**): Averaged RMSD values (Å) with standard deviation over 20 ns of MD simulation for ligand-interacting residues in wild-type Tyr (blue squares) and the P406L mutant variant (red circles). Statistical significance was assessed using a two-tailed Student’s t-test based on RMSD values calculated at 1 ns intervals across the 20 ns simulation for each protein. Statistical significance levels are indicated as follows: *p* < 0.05 (*), *p* < 0.01 (**), and *p* < 0.001 (***). Panel (**C**): Per residue number of contacts for Tyr ligand-interacting residues in Tyr (blue) and the P406L mutant variant (pink). Scale bars range from 0 (white, no contacts) to 70 (deep blue or pink, 13 contacts). Panel (**D**): ΔDCCM (DCCMP406L–DCCMTyr) of Tyr ligand-interacting residues, highlighting changes in dynamic correlations. The scale bar indicates positive (blue) and negative (pink) correlations. Panel (**E**): Averaged SASA over 20 ns of MD simulation for Tyr (**a**) and the P406L mutant (**b**). Color intensity reflects the ratio of side-chain surface area to the random coil value per residue, ranging from 0% (red, most buried) to 100% (blue, most exposed).

The P406L mutation markedly disrupted active site organization and coordinated behavior. Several residues that normally participate in substrate binding (E345, F347, A357, N364, H367, I368, M374, S375, F386, H390) lost their secondary structure, indicating reduced structural integrity (Figure 4A and Figure S3).

Conversely, H363, G379, and S380 gained α- or 310-helical structure, suggesting localized structural stabilization of regions that require flexibility for substrate accommodation.

Although catalytic residues remained comparatively stable, many peripheral and loop-associated residues (F347, M374, S375, F386) displayed increased RMSD, indicating local destabilization in areas essential for substrate binding (Figure 4B). Contact number decreased in 70% of the residues (Figure 4C and Figure S4), and 74% showed increased SASA (Figure 4E and Figure S5), reflecting weakened packing and increased solvent exposure. DCCM analysis further revealed decreased positive correlation in 91% of residues (Figure 4D and Figure S6), consistent with disruption of the coordinated motions that support catalytic alignment.

Together, these data show that P406L compromises local and global stability, disrupts active site communication, and impairs the dynamic plasticity required for efficient substrate processing.

### 2.6. Effect of ClinVar-Listed Mutations on Active Site Dynamics

**Figure 5:**
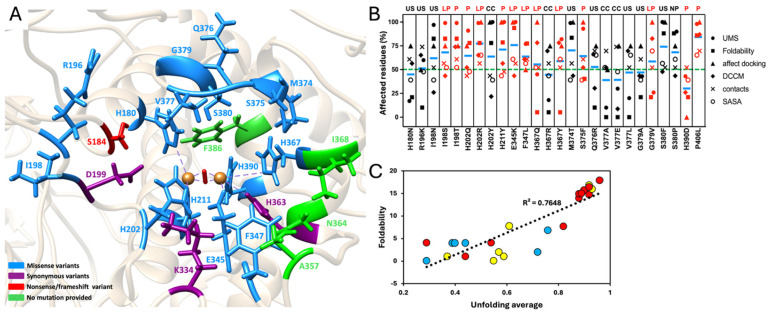
Effect of ClinVar-listed mutations on Tyr’s active site dynamics. Panel (**A**): Homology model of the Tyr intra-melanosomal domain showing ClinVar-listed mutations in Tyr’s ligand-interacting residues identified as missense variants (blue), synonymous variants (purple), and nonsense/frameshift variants (red). Residues without reported mutations are depicted in green. The protein backbone is shown as a tan ribbon, and copper atoms in the active site, represented as orange spheres, are stabilized by an O_2_ molecule positioned between them and represented as a red rod. Panel (**B**): Percentage of Tyr’s ligand-interacting residues affected by ClinVar-listed mutations, based on multiple computational and dynamic analyses. Each symbol represents a distinct type of mutation-associated change: predicted UMS (circle), foldability (square), affected docking (triangle), DCCM changes (rhombus), changes in per residue number of contacts (cross), and increasing SASA (open circle). Red symbols indicate pathogenic or likely pathogenic variants. Blue dashes represent the grand means for each mutant. The green dashed line marks the 50% threshold. Top panel abbreviations refer to ClinVar classifications: US, uncertain significance; LP, likely pathogenic; P, pathogenic; CC, conflicting classifications of pathogenicity; NP, not provided. Panel (**C**): Correlation between foldability and unfolding average across residues. A scatter plot showing the linear relationship (dashed line) between calculated foldability and unfolding average values (NEI Commons Ocular Proteomes website), with an overall correlation of R^2^ = 0.76. Residues carrying mutations classified by ClinVar as pathogenic or likely pathogenic are colored red, those classified as benign/likely benign are colored blue, and those with conflicting/uncertain classification are colored yellow.

We evaluated the impact of 25 ClinVar-listed missense variants affecting 23 interacting residues using unfolding mutation screen (UMS), foldability analysis, molecular docking, DCCM, per residue contact analysis, and SASA (Figure 5, Table S2, Figure S7). Each mutant was analyzed using a single 20 ns MD trajectory under identical simulation conditions.

Mapping all ClinVar variants onto the Tyr intra-melanosomal domain showed that missense, synonymous, and nonsense/frameshift mutations are distributed across the ligand-interacting residues, including positions directly involved in anchoring, triad formation, and loop dynamics (Figure 5A). Across the majority of mutants, particularly those classified as pathogenic or likely pathogenic, over 50% of substrate-interacting residues exhibited significant perturbations in at least one structural or dynamic parameter, indicating broad disruption of the active-site environment (Figure 5B). Typical effects included reduced contacts, loss of secondary structure, increased solvent exposure, or diminished correlated motions, features that collectively reflect impaired stability and weakened residue-residue communication (Figure S7). Disruptions were especially frequent within the 374–386 gating loop and the anchoring residues (K334, E345, F347, H363, N364, H367).

Foldability scores and unfolding averages for residues carrying ClinVar mutations, obtained from the NEI Commons Ocular Proteomes resource, agreed (R^2^ = 0.76), especially for mutant variants classified as pathogenic or likely pathogenic (Figure 5C).

Overall, ClinVar-listed mutations frequently destabilize the structural and dynamic framework surrounding the active site and perturb the networks required to maintain a catalytically competent conformation.

## 3. Discussion

### 3.1. Molecular Basis of Human Tyr Multi-Substrate Plasticity

Understanding how Tyr, an enzyme operating at the entry point of melanogenesis, dynamically organizes its active site to process chemically distinct substrates has remained unresolved. Here, we address this gap through a comprehensive computational strategy that maps active site behavior across all major melanogenic intermediates. Docking and MD analyses identified 23 residues that define the Tyr active site interaction set, comprising universal anchoring positions and ligand-specific contacts that together shape substrate binding (Figure 1, Table S1). Notably, all residues except A357 are evolutionarily conserved, highlighting that the core architecture used for substrate recognition and catalytic alignment has been preserved under strong evolutionary pressure, reinforcing the functional importance of these positions (Figure 2).

The chemical and structural principles underlying this multi-substrate plasticity are summarized in Table 1, which integrates the substrate’s chemical properties, functional role in melanogenesis, specific interacting residues, universal anchoring positions, and relative copper engagement. This integrative view highlights how substrate polarity, charge, aromaticity, and redox activity are translated into distinct interaction patterns within the active site.

The substrate-specific interactions follow clear structural and chemical principles: each ligand engages the region of the active site that best accommodates its shape, polarity, and/or position in the melanogenic pathway (Figure 1, Table 1). L-tyrosine, the smallest and least redox-active precursor [1,3], engages H180 and G379 in the CuA-proximal region that initiates the first monophenol oxidation step [24,25]. L-DOPA, a more polar and redox-active catechol, interacts with A357 and CuB-coordinating H363, reflecting the need for precise diphenol alignment during rapid oxidation [8,24]. DHICA, the only negatively charged substrate, uniquely recruits the positively charged R196 through electrostatic complementarity; however, this positioning does not direct DHICA toward the CuA/CuB center, consistent with its known preference for oxidation by Tyrp1 rather than Tyr [26]. DHI, the most aromatic and redox-active intermediate, engages I368 and N364 along the lateral catalytic wall, where Tyr can transiently stabilize this highly reactive indole and prevent uncontrolled self-oxidation during the final stages of eumelanin formation [8,26].

Despite these ligand-specific differences, all four substrates converge on a shared anchoring triad (H367, F347, S375), highlighting a universal recognition mechanism centered on the CuB-proximal region and the 374–386 gating loop (Figure 1, Table 1). This dual strategy, universal anchoring combined with substrate-specific adjustment, may explain how Tyr accommodates substrates with distinct sizes, charges, and redox properties within a single catalytic site.

MD-based analyses showed that substrate engagement stabilizes the catalytic architecture and reorganizes key dynamic networks (Figure 3). Across all substrates, more than half of the interacting residues exhibit stabilization in at least one metric (RMSD, RMSF, DCCM, contacts, SASA), indicating that ligand binding promotes coordinated structural adjustments that reinforce the active site integrity (Figure S8).

From our analysis, we might expect that the binding of the initial substrate, L-tyrosine, promotes classical stabilization of the active-site histidines and surrounding framework, accompanied by moderate adjustment in nearby loops that facilitate substrate entry. This configuration appears optimized for catalytic activation and initiation of the melanogenic cascade. As the pathway progresses, L-DOPA, being larger and more polar, induces more pronounced loop remodeling, with these structural shifts propagating through long-range dynamic coupling. DHICA, although recognized, triggers substantial rearrangements at the channel entrance, rather than within the deeper catalytic cavity, consistent with its weaker catalytic engagement and its downstream routing to Tyrp1 later in the pathway. DHI, despite its intrinsic tendency to auto-oxidize, strongly stabilizes the catalytic core while promoting adaptive loop remodeling, including transient helix formation and contact rewiring, changes likely required to control its high reactivity before eumelanin polymerization.

Importantly, two independent layers of analysis, substrate-specific interacting residues and MD-based stabilization metrics, point to the same principle: Tyr responds to each substrate in a manner that reflects the substrate’s chemistry and its functional role in the melanogenic pathway. As summarized in Table 1, early monophenolic substrates preferentially occupy CuA-proximal regions, whereas more oxidized intermediates display increased CuB or combined dicopper engagement. This agreement between binding geometry, copper coordination patterns, and dynamic behavior reinforces an ordered pattern of Tyr substrate recognition.

### 3.2. Impact of Pathogenic Variants on Active-Site Dynamics

This finely tuned conformational adaptability is susceptible to disruption. The OCA1B-associated P406L mutation, although located far from the catalytic site, exerts a pronounced allosteric effect on the active site network. P406L reduces positively correlated motion in 87% of substrate-binding residues, decreases intramolecular contacts, and increases solvent exposure (Figure 4 and Figure S8). Residues critical for ligand accommodation and gating, F347, M374, S375, and F386, are particularly destabilized, indicating impaired control of the substrate entry corridor. In parallel, H363, G379, and S380 gain helical structure, reflecting a shift toward more rigid local conformations that limit the flexibility normally required for substrate positioning and loop remodeling. Together, these changes might suggest that P406L disrupts Tyr function not by perturbing catalytic residues directly, but by weakening long-range dynamic coupling and restricting the adaptive motions essential for multi-substrate recognition. This mechanism is fully consistent with P406L’s established association with OCA1B [25,27,28].

ClinVar-listed mutations at substrate-interacting residues show a consistent pattern of disruption. Across most missense variants, especially those classified as pathogenic or likely pathogenic, more than half of substrate-interacting residues display a substantial shift in docking scores, DCCM values, number of contacts, or UMS (Figure 5B and Figure S7). These changes imply weakened anchoring, altered flexibility, and compromised residue–residue communication. In addition, foldability and unfolding metrics aligned closely for residues carrying ClinVar variants, especially pathogenic ones, highlighting their intrinsic vulnerability to destabilization (Figure 5C). Although each variant was analyzed using a single 20 ns trajectory to enable systematic comparison across many mutants, longer and replicated simulations would allow more extensive conformational sampling and further refine estimates of mutation-dependent dynamic variability. Taken together, these results indicate that many clinically observed Tyr variants disrupt active-site dynamics and coupling, providing mechanistic explanations for pigmentation phenotypes such as oculocutaneous albinism.

### 3.3. Genetic Mutation: Sensitive Regions in the Catalytic Site

**Figure 6:**
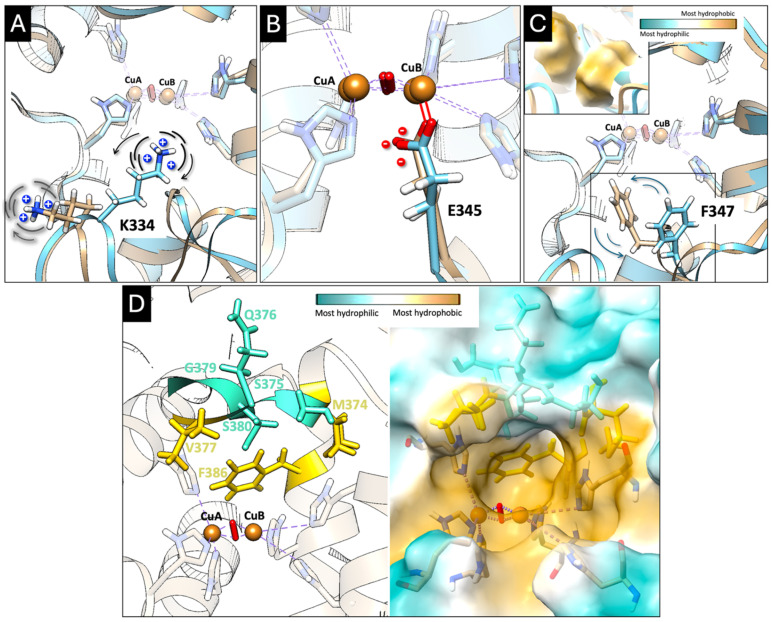
Consistently responsive residues across all structural and dynamic analyses. Potential electrostatic and hydrophobic anchoring zone: K334 (substrate anchoring and electrostatic steering, Panel (**A**)), E345 (copper-linked electrostatic modulator, Panel (**B**)), and F347 (hydrophobic gatekeeper in tunnel dynamics, Panel (**C**)). The protein backbone is shown as a ribbon, colored tan at 0 ns and blue at 20 ns of MD simulation. Copper atoms (CuA and CuB) in the active site are represented as orange spheres, and the bridging O_2_ molecule is shown in red. The red line indicates the ionic/ion-dipole coordination between E345 and CuB. Panel (**D**) shows loop 374–386 as the substrate accommodation and dynamic adaptation module. Hydrophobic residues are colored yellow and hydrophilic residues cyan (**left**). The protein backbone is shown as a tan ribbon, and copper atoms (CuA and CuB), represented as orange spheres, are stabilized by an O_2_ molecule represented as a red rod. The Tyr surface is colored according to hydrophobicity (bar scale: cyan—most hydrophilic; yellow—most hydrophobic) (**right**).

To understand how human Tyr responds to sequence variation, we first defined mutation sensitivity as the degree to which a residue exhibits destabilization: loss of contacts, increased solvent exposure, elevated RMSD and RMSF, or reduced correlated motion when mutated. Residues 334–347 were ~1.7-fold more susceptible to disruption upon mutation than residues 374–386, underscoring both their structural sensitivity and their potential value as engineering targets (Figure S9). Within the anchoring zone, K334, E345, and F347 participate directly in the initial docking interactions with substrates, and function as a coordinated electrostatic/hydrophobic module: K334 provides positive electrostatic steering and becomes more ordered upon binding, E345 contributes a partially solvent-exposed negative framework that enhances allosteric coupling, and F347 acts as a hydrophobic gatekeeper that shapes the access tunnel and modulates substrate entry (Figure 6A–C).

A second responsive region, the 374–386 gating loop, represents a substrate accommodation module (Figure 6D). Hydrophilic and polar residues (S375, Q376, S380, G379) provide flexibility and dynamic coordination, while hydrophobic or bulky residues (M374, V377, F386) ensure transient gating and local stability. This loop exhibits elevated RMSD, RMSF, and DCCM changes, consistent with a mobile gating mechanism that transiently opens to admit substrates and then reshapes to stabilize the bound state.

Using this framework, the dynamic metrics derived here provide a basis for predicting how individual missense variants disrupt active site stability and catalytic alignment. These analyses also highlight regions with strong potential for rational protein engineering. Together, these two modules define a tunable dynamic architecture that guides substrate orientation and catalytic readiness, revealing clear engineering targets for modifying substrate specificity, turnover rate, or stability. Furthermore, dynamic metrics such as DCCM, UMS, contact maps, and foldability scores provide complementary tools for interpreting variants of uncertain significance by linking sequence changes to disruptions in active site communication.

### 3.4. Tyrosinase Ordered Binding Mechanism

**Figure 7:**
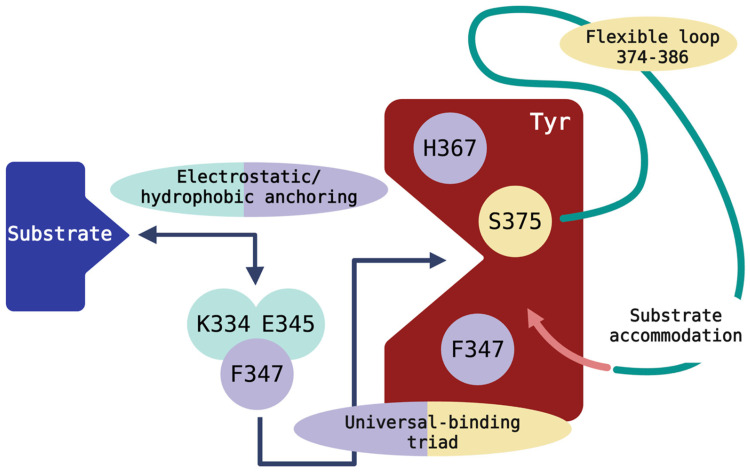
Proposed ordered binding mechanism in Tyr. The schematic depicts three residue clusters proposed from MD-derived interaction patterns: an anchoring module (K334, E345, or F347), a substrate-universal triad (H367, F347, S375), and the 374-386 accommodation loop. Residues appearing in overlapping regions (F347, S375) are predicted to contribute to multiple functional roles.

Analysis of our computational simulations supports an ordered binding mechanism for Tyr’s multi-substrate recognition (Figure 7).

In this proposed model, substrates first encounter an electrostatic/hydrophobic anchoring zone (K334, E345, F347), that directs them toward the catalytic pocket. They then converge on a universal binding triad (H367, F347, S375), which positions all ligands next to the CuB site. Finally, movement of the 374-386 gating loop enables ligand-specific contacts, H180 and G379 for L-tyrosine, A357 and H363 for L-DOPA, R196 for DHICA, and I368 and N364 for DHI, to refine insertion depth, orientation, and copper engagement according to each substrate’s chemistry and biosynthetic context.

In this model, Tyr does not depend on a fixed catalytic geometry; instead, it combines a conserved anchoring core with a flexible accommodation loop to create substrate-specific binding modes while preserving catalytic accuracy. This organized binding framework explains how Tyr can process chemically diverse substrates despite deep evolutionary conservation and why disruptions in anchoring residues, the universal triad, or loop flexibility hinder catalysis. It also offers a mechanistic understanding for interpreting mutation effects and highlights engineering opportunities within Tyr’s anchoring and gating regions. These insights support the rational design of Tyr variants with altered catalytic properties and the development of structure-guided inhibitors or activators relevant to pigmentation disorders.

To our knowledge, this ordered binding mechanism, combining an anchoring zone, a universal recognition triad, and a substrate-specific accommodation loop, has not been previously described for human Tyr or for enzymes in the pigmentation pathway at this level of substrate resolution. Substrate promiscuity and loop gating are well documented in several enzyme families [29,30,31]; however, the present work identifies a substrate-resolved and residue-specific implementation of these principles in human Tyr, integrating anchoring, universal alignment, and loop-mediated refinement within a single catalytic framework.

It is important to note that the proposed ordered binding mechanism and the associated anchoring region are delivered from in silico analyses and therefore represent model-supported mechanistic inferences rather than experimentally validated molecular events. While the simulations reveal reproducible substrate-dependent interaction patterns and dynamic responses consistent with known biochemical properties of Tyr substrates, direct experimental validation by site-directed mutagenesis or biochemical assays has not yet been performed. Accordingly, this framework should be viewed as a hypothesis-generating model that delineates testable principles of Tyr substrate recognition and active site organization.

All dynamic conclusions presented here should be interpreted as model-supported predictions derived from molecular simulations, rather than as established mechanistic facts. The 20 ns simulation timescale was selected to assess relative ligand stability and active-site interaction patterns, and longer simulations will be required to capture slower conformational rearrangements. While preliminary experimental analyses of ligand binding reported previously [18] support the relevance of the identified interaction regions, extended simulations and targeted biochemical validation will be essential to further refine and test these predictions.

The ability to link specific mutations to changes in dynamic coupling, residue interactions, and substrate engagement also opens new opportunities for functional evaluation of Tyr missense variants in clinical genomics. Structural and dynamic metrics derived from our simulations, including DCCM or UMS, may aid in classifying uncertain variants and refining genotype–phenotype predictions in pigmentation disorders. However, these computational findings now require experimental validation. Future studies combining targeted mutagenesis, biochemical assays, and structural approaches will be essential to validate and refine the predicted anchoring interactions, triad positioning, and loop dynamics, and to further strengthen the proposed binding mechanism.

## 4. Materials and Methods

### 4.1. Framework for Active Site Mapping

Active-site mapping was performed by integrating molecular docking with molecular dynamics (MD) simulations to identify 23 residues that consistently might participate in ligand recognition, as summarized in Supplementary Table S2. Docking provided initial ligand poses, and a preliminary contact set as defined in the Method’s paragraph 3. Residues showing persistent interactions across ligand complexes and simulations were classified as key interacting residues, providing a dynamically informed definition of the Tyr active site.

### 4.2. Homology Modeling and MD Simulations of Tyr and Its Mutant Variants

A homology model of recombinant human Tyr (residues 19–469) was built in YASARA (http://www.yasara.org (accessed on 20 May 2025)) [32] using the crystal structure of human Tyrp1 (PDB ID: 5M8L, https://www.rcsb.org/structure/5M8L (accessed on 5 February 2019)) as a structural template [33]. The model was glycosylated using GLYCAM-Web (http://glycam.org (accessed on 5 February 2019)) as previously described [34]. To accurately represent the dicopper active site, coordination bonds between the copper ions and the coordinating histidine residues were modeled as pseudo-bonds, thereby maintaining experimentally supported bond lengths and coordination geometry throughout the simulations. Partial charges for the copper ions were automatically assigned using the YASARA AutoSMILES procedure. Before MD simulations, the geometry of the active-site copper cluster was optimized by initially positioning both copper ions approximately 3 Å from their coordinating histidine residues, followed by semiempirical quantum mechanical optimization using MOPAC 7. Implicit solvation was included during this optimization using the COSMO model. The resulting structural model was examined for stereochemical quality and structural integrity using the SAVES validation server (https://www.doe-mbi.ucla.edu/services/ (accessed on 15 June 2022)) [19]. Point mutations for all analyzed Tyr mutant variants were introduced using the Edit > Swap > Residue function in YASARA. All structures underwent energy minimization before being subjected to 20 ns MD simulations using YASARA’s default run.mcr macro. Simulations were run under standard conditions: temperature, 298 K; pH, 7.4; water density, 0.997 g/mL; and 0.9% NaCl as a mass fraction. A cubic simulation cell was generated with 5 Å clearance around the protein, resulting in dimensions of 92.84 Å × 92.84 Å × 92.84 Å. Simulation snapshots were saved every 0.25 ns, resulting in 80 frames per 20 ns trajectory.

The homology model and simulation framework used in the present study were previously validated through extended 100 ns MD simulations of wild-type and mutant tyrosinase variants, which demonstrated stable RMSD plateau behavior and structural robustness of the catalytic core [35]. The same modeling strategy was employed here to ensure methodological consistency.

### 4.3. Ligand Preparation and Docking Simulations

The 3D structures of the small-molecule ligands (L-tyrosine, L-DOPA, DHICA, and DHI) were retrieved in SDF format from PubChem (http://pubchem.ncbi.nlm.nih.gov (accessed on 31 October 2023)) and converted to PDB format using UCSF Chimera (v1.16.0, UCSF, San Francisco, CA, USA) [36]. Ligands were energy-minimized using the AMBER14 force field.

Molecular docking simulations were performed in a vacuum and water environment using VINA implemented in YASARA (v22.9.24, IMBM, University of Graz, Graz, Austria). The dock_run.mcr macro was used to execute docking. Each ligand underwent 25 docking runs, and results were ranked by binding energy (kcal/mol) and estimated dissociation constant (pM). Docked poses differing by at least 5.0 Å in heavy-atom root mean square deviation (RMSD) were clustered. Ligand torsion parameters and atomic charges were derived from the AMBER03 force field [37]. Ligands were considered properly docked if the oxygen atom on their aromatic ring was oriented toward the binuclear copper site of Tyr. Additionally, docking was accepted as valid when the distance between a ligand’s hydroxyl or carbonyl oxygen and the CuA or CuB atoms was ≤4.0 Å.

All validated ligand-bound Tyr or mutant variant structures were subjected to 20 ns MD simulations carried out using the AMBER14 force field and the runfast.mcr macro in YASARA, which includes automatic equilibration steps following energy minimization. MD simulations were performed to assess the stability of docked ligand binding within the catalytic site. Snapshots were output every 0.1 ns. Complexes were analyzed at 1 ns intervals over the 20 ns trajectory, and alignments were evaluated.

A complex was defined as valid if the ligand maintained its position near the copper site (≤4.0 Å deviation) throughout the simulation. Using this classification, we selected and further analyzed 3 poses for L-tyrosine, 2 poses for L-DOPA, 7 poses for DHICA, and 6 poses for DHI.

Protein–ligand interactions, including hydrophobic and π–π stacking, were identified in YASARA if the interacting atoms were within 5 Å and not bonded. Hydrogen bonds were further analyzed in UCSF Chimera using relaxed constraints (0.4 Å tolerance, 20° angle cutoff). Docked complexes were visualized in Chimera.

### 4.4. Analysis of MD Trajectories

MD trajectories for wild-type Tyr, Tyr in complex with substrates, and all analyzed ClinVar mutant variants (H180N, R196K, I198N, I198S, I198T, H202Q, H202R, H202Y, H211Y, E345K, F347L, H367Q, H367R, H367Y, M374,T, S375F, Q376R, V377A, V377E, V377L, G379A, G379V, S380F, S380P, H390D, and P406L) were processed using md_analyze.mcr in YASARA (24.4.10.M.64) over a period of 20 ns with 81 snapshots and the AMBER14 force field. For ClinVar-listed mutant variants, a single 20 ns MD trajectory was analyzed for each mutant. For wild-type Tyr and the P406L mutant, three independent time-window analyses were performed to assess the reproducibility of dynamic trends. For substrate-bound systems, only poses that remained stable during MD simulations were retained for further analysis, corresponding to three Tyr/L-tyrosine complexes, two Tyr/L-DOPA complexes, seven Tyr/DHICA complexes, and six Tyr/DHI complexes. From these trajectories, the following structural and dynamic descriptors were extracted: secondary structure per residue, per residue number of contacts, root mean square fluctuation (RMSF) per residue, and dynamic cross-correlation matrices (DCCMs).

### 4.5. Root Mean Square Deviation (RMSD)

For the wild-type Tyr and the P406L variant, per residue RMSD values were calculated every 1 ns over the 20 ns trajectory and averaged. For per residue RMSD calculations, two molecular structures (reference and simulated) were first superimposed in YASARA using Cα atoms for structural alignment. Then, the RMSD of individual residues was computed across the trajectory using the Analyze > RMSD per residue function in YASARA.

### 4.6. Solvent-Accessible Surface Area (SASA)

Per residue SASA was calculated using GetArea (https://curie.utmb.edu/getarea.html (accessed on 30 June 2025)) [38] with a 1.4 Å water probe radius. SASA was analyzed for unbound Tyr and the P406L mutant, as well as for Tyr bound to L-tyrosine, L-DOPA, DHICA, and DHI. Values were calculated at 1 ns intervals over a 20 ns simulation and then averaged to assess residue-specific solvent exposure throughout the trajectory. For ClinVar-listed mutant models, SASA was calculated for the 20 ns structures.

Values for SASA, RMSD, RMSF, secondary structure, residue contacts, and DCCM were extracted specifically for the interacting residues involved in substrate binding and catalytic activity. These interacting residues were identified based on contact analyses from docking and were used as the focus for all comparative evaluations across wild-type, mutant, and substrate-bound systems.

### 4.7. The Unfolding Mutation Screen (UMS) and Foldability

The full-atomic Tyr homology model and the results of the UMS and foldability are freely available from the ocular proteome website at the NEI Commons Ocular Proteomes website (https://neicommons.nei.nih.gov/#/proteomeData (accessed on 20 March 2025)). UMS values (ranging from 0 to 1) were normalized and are expressed as percentages (1 = 100%) to aid in comparative analysis [34,39]. Foldability values are expressed as a percentage, using 19 as the maximum reference value.

### 4.8. Protein Sequence Alignment Across Species

Protein sequences from human, chicken, mouse, gorilla, bovine, cat, dog, fungi (*Aspergillus*, *Neurospora*), and bacteria (*Streptomyces*) were retrieved from the UniProtKB database and aligned using the UniProt multiple sequence alignment tool (https://www.uniprot.org/align (accessed on 10 June 2025)). Residue conservation was defined based on positional identity across the aligned sequences, with conservation assessed within vertebrates and across more distantly related species.

### 4.9. Quantification of Mutation-Induced Destabilization in Active Site Residue Groups

To evaluate mutation susceptibility within defined regions of the catalytic site, two residue groups were analyzed: residues 334–347 (K334, E345, F347) and residues 374–386 (M374, S375, Q376, V377, G379, S380, F386). The destabilizing effect of ClinVar-listed mutations was quantified relative to the wild-type Tyr structure. Differences between mutant and wild-type values were calculated for structural and dynamic parameters, including RMSD, RMSF, SASA, intramolecular contacts, and DCCM. Increases in RMSD, RMSF, and SASA were considered destabilizing, whereas decreases in contacts and DCCM were interpreted as loss of structural coupling and thus destabilizing. Mutation-induced changes were averaged across all analyzed variants at the residue level, and residue-level means were subsequently averaged within each group to generate a group-level disruption score. The relative susceptibility to mutation was determined by calculating the ratio of the two group-level scores, yielding the reported ~1.7-fold difference.

## 5. Conclusions

Our results demonstrate that human Tyr relies on distinct, substrate-specific conformational responses to regulate catalysis, and that disease-associated mutations disrupt this regulation through long-range allosteric effects. By establishing a dynamic, simulation-based map of the active site, we reveal possible transient conformations and a communication network that are difficult to capture with static structural methods. We uncover dynamic states that are not captured by static techniques and remain challenging to resolve experimentally with residue-level detail. Because similar dynamic principles underlie the function of many enzymes, this framework provides a broadly applicable foundation for mechanistic annotation, interpretation of missense variants, and rational design of engineered enzymes with tailored catalytic properties.

## Figures and Tables

**Table 1:** Comparative analysis of melanogenic substrates and their interaction patterns within the human Tyr active site.

	L-tyrosine	L-DOPA	DHICA	DHI
Key chemical properties	Amphipathic monophenolic amino acid with a flexible aliphatic side chain, moderate polarity, and low intrinsic redox activity	Hydrophilic catechol amino acid with high polarity and high redox activity	Negatively charged carboxylated dihydroxyindole with strong hydrophilic characteristic, high polarity, and moderate redox activity	Neutral indole with a rigid fused ring system, reduced polarity, and high radical reactivity
Primary role in melanogenesis	Initial substrate; precursor to L-DOPA	Central intermediate; branching point toward eumelanin or pheomelanin	Major precursor of DHICA-rich eumelanin	Precursor of DHI-rich eumelanin; promotes aggregation
Reaction catalyzed by Tyr	Monophenolase activity: hydroxylation to L-DOPA	Diphenol oxidase activity: oxidation to dopaquinone	Oxidation to IQCA	Oxidation to IQ
Substrate-specific interacting residues	H180, G379	A357, H363	R196	N364, I368
Chemical complementarity (substrate’s property/interacting residue)	Polar (H180) and neutral (G379) interactions may contribute to stabilization of initial monophenol positioning near the active site entrance	Hydrogen bonding and polar contacts may accommodate catechol hydroxyls	Electrostatic interactions with the positively charged R196 may contribute to stabilization of the negatively charged carboxylate	Polar (N364)/hydrophobic (I368) balance may stabilize the indole ring and π-stacking interactions
Common interacting residues (universal triad)	F347, H367, S375			
Recognition of the universal triad	Aromatic stacking with the phenyl ring and adaptable polar contacts may enable anchoring of the monophenolic aromatic ring	Enhanced hydrogen bonding and aromatic interactions may stabilize the catechol aromatic ring within the common aromatic polar platform	Combined aromatic stacking and polar interactions may accommodate the indole ring system while tolerating the carboxylate substitution	Aromatic stacking and minimal polar constraints may stabilize the rigid indole ring system within the shared anchoring framework
Relative interaction with copper center	Interacts primarily with residues proximal to CuA, consistent with shallow binding during the initial monophenolase stage	Interacts with A357 and the CuB-coordinating H363, consistent with engagement of the CuB environment that supports precise diphenol alignment during oxidation	Exhibits limited CuA/CuB engagement, as electrostatic anchoring via R196 favors peripheral positioning, consistent with controlled oxidation and restrained polymer growth	Engages both CuA and CuB, consistent with radical-driven polymer growth characteristic of DHI-rich eumelanin

## Data Availability

Data available upon request.

## Supplementary Materials

The following supporting information can be downloaded at https://www.mdpi.com/article/10.3390/ijms27041937/s1.

## Abbreviations

The following abbreviations are used in this manuscript:

Tyr	Human recombinant tyrosinase
OCA1	Oculocutaneous albinism type 1
OCA1A	Oculocutaneous albinism type 1A
OCA1B	Oculocutaneous albinism type 1B
P406L	OCA1B-associated mutation, P406L
Tyrp1	Tyrosinase-related protein 1
Tyrp2	Tyrosinase-related protein 2
DHICA	5,6-dihydroxyindole-2-carboxylic acid
DHI	5,6-dihydroxyindole
MDs	Molecular dynamics simulations
RMSD	Root mean square deviation
RMSF	Root mean square fluctuation
DCCM	Dynamic cross-correlation matrices
SASA	Solvent accessible surface area
UMS	Unfolding mutation screen
CuA	Copper atom A
CuB	Copper atom B
