# Supplementary material for: Insights from Computational Dynamic Active Site Mapping into Substrate Recognition and Mutation-Induced Dysfunction in Human Tyrosinase
Source: Int J Mol Sci. 2026 Feb 18;27(4):1937. doi: 10.3390/ijms27041937 (PMC12940576; doi:10.3390/ijms27041937)
Supplement: Supplementary file 1 [file ijms-27-01937-s001.zip › ijms-4129480-supplementary.pdf]

Supplementary Material

Table S1. Key residues involved in small-molecule substrates binding to Tyr during molecular dynamic (MD) simulations.

	L-tyrosine			L-DOPA			DHICA			DHI		
	HB	HpC	π-π	HB	HpC	π-π	HB	HpC	π-π	HB	HpC	π-π
**Before MD**	K334	K334<br>H367<br>V377	H202	N364	F347<br>H367	H367	I198<br>D199<br>K334<br>E345	E345<br>F347<br>H367<br>V377	H202<br>H367	E345<br>S380	F347<br>H367<br>V377	H367
**After MD**	H180<br>S184<br>D199<br>M374<br>S375<br>Q376<br>V377<br>G379<br>S380	H180<br>S184<br>F347<br>H367<br>S375<br>V377<br>S380<br>F386	H180<br>F347<br>H367<br>F386	A357<br>S375<br>Q376<br>S380	H367<br>S380	H363<br>H367	R196<br>D199<br>K334<br>S375<br>V377	S184<br>D199<br>H202<br>E345<br>F347<br>H367<br>V377	H202<br>F347<br>H367	N364<br>M374<br>S375<br>Q376<br>V377<br>S380	F347<br>H367<br>I368<br>V377<br>S380	H202<br>F347<br>H367
**Total**	H180<br>S184<br>D199<br>H202<br>K334<br>F347<br>H367<br>M374<br>S375<br>Q376<br>V377<br>G379<br>S380<br>F386			H363<br>N364<br>F347<br>A357<br>H367<br>S375<br>Q376<br>S380			S184<br>R196<br>I198<br>D199<br>H202<br>K334<br>E345<br>F347<br>H367<br>S375<br>V377			H202<br>E345<br>F347<br>N364<br>H367<br>I368<br>M374<br>S375<br>Q376<br>V377<br>S380		

HB, hydrogen bonds; HpC, hydrophobic contacts; π–π, Pi–Pi stacking; Substrate-specific residues are shown in red, residues common to all substrates are shown in green, and all other interacting residues are shown in black.

Table S2. ClinVar listed mutations in Tyr's ligand-interacting residues.

Residue	Protein Change	Single nucleotide variant type	Variation	Condition*	Classification*	Unfolding average**	Predicted UMS**	Foldability**
**H180**	H180N	missense variant	538C>A (p.His180Asn)	not specified	**Uncertain significance**	0.39	0.17	3.98
	n/a	synonymous variant	540T>C (p.His180=)	not specified	**Likely benign**		n/a	
**S184**	S184fs	frameshift variant	549del (pSer184fs)	OCA1B	**Likely pathogenic**	0.88	n/a	13.88
	S184*	Nonsense	551C>G (pSer184Ter	light/dark skin	**Pathogenic**		n/a	
**R196**	R196K	missense variant	587G>A (p.Arg196Lys)	not provided	**Uncertain significance**	0.72	0.6	1.93
	n/a	synonymous variant	586A>C (p.Arg196=)	not provided	**Likely benign**		n/a	
**I198**	I198N	missense variant	593T>A (p.Ile198Asn)	not provided	**Uncertain significance**	0.9	0.97	15.68
	I198T	missense variant	593T>C (p.Ile198Thr)	OCA1A/B	**Pathogenic/Likely pathogenic**		0.99	
	I198S	missense variant	593T>G (p.Ile198Ser)	not provided	**Likely pathogenic**		0.99	
**I199**	n/a	synonymous variant	597T>C (p.Asp199=)	not provided	**Likely benign**	0.4	n/a	3.93
**H202**	H202Q	missense variant	606T>G (p.His202Gln)	OCA1A/B	**Pathogenic/Likely pathogenic**	0.88	0.91	14.83
	H202R	missense variant	605A>G (p.His202Arg)	not provided	**Likely pathogenic**		0.99	
	H202Y	missense variant	604C>T (p.His202Tyr)	OCA1A/B	**Conflicting classifications**		1	
	n/a	synonymous variant	606T>C (p.His202=)	not provided	**Likely benign**		n/a	
**H211**	H211Y	missense variant	631C>T (p.His211Tyr)	not provided	**Pathogenic**	0.89	1	14.93
	n/a	synonymous variant	633C>T (p.His211=)	not provided	**Likely benign**		n/a	
**K334**	n/a	synonymous variant	1002A>G (p.Lys334=)	not provided	**Likely benign**	0.76	n/a	6.76
**E345**	E345K	missense variant	1033G>A (p.Glu345Lys)	not provided	**Likely pathogenic**	0.96	1	17.83
**F347**	F347L	missense variant	1039T>C (p.Phe347Leu)	OCA1A	**Likely pathogenic**	0.92	0.77	14.72
**A357**	n/a	n/a	n/a	n/a	n/a	0.55	n/a	0.00
**H363**	n/a	synonymous variant	1089C>T (p.His363=)	not provided	**Likely benign**	0.44	n/a	3.91
**N364**	n/a	n/a	n/a	n/a	n/a	0.37	n/a	1.00
**H367**	H367Q	missense variant	1101C>A (p.His367Gln)	OCA1A	**Likely pathogenic**	0.44	0.45	0.99
	H367R	missense variant	1100A>G (p.His367Arg)	OCA1A	**Conflicting classifications**		0.18	
	H367Y	missense variant	1099C>T (p.His367Tyr)	OCA1A	**Likely pathogenic**		0.82	
**I368**	n/a	n/a	n/a	n/a	n/a	0.59	n/a	0.97
**M374**	M374T	missense variant	1121T>C (p.Met374Thr)	not provided	**Uncertain significance**	0.93	0.99	15.87
**S375**	S375F	missense variant	1124C>T (p.Ser375Phe)	not provided	**Pathogenic**	0.82	0.93	7.66
	n/a	synonymous variant	1125C>G (p.Ser375=)	not provided	**Likely benign**		n/a	
**Q376**	Q376R	missense variant	1127A>G (p.Gln376Arg)	Inborn genetic diseases	**Uncertain significance**	0.57	0.66	1.93
**V377**	V377A	missense variant	1130T>C (p.Val377Ala)	not provided	**Conflicting classifications**	0.29	0.1	0
	V377E	missense variant	1130T>A (p.Val377Glu)	OCA1A/B	**Conflicting classifications**		0.3	
	V377L	missense variant	1129G>T (p.Val377Leu)	not provided	**Uncertain significance**		0.2	
	n/a	synonymous variant	1131A>G (p.Val377=)	not provided	**Likely benign**		n/a	
**G379**	G379A	missense variant	1136G>C (p.Gly379Ala)	not provided	**Uncertain significance**	0.54	0.43	4.00
	G379V	missense variant	1136G>T (p.Gly379Val)	Ligh/dark skin	**Likely pathogenic**		0.26	
**S380**	S380F	missense variant	1139C>T (p.Ser380Phe)	not provided	**Uncertain significance**	0.92	1	16.75
	S380P	missense variant	1138T>C (p.Ser380Pro)	not provided	**not provided**		0.9	

**F386**	n/a	n/a	n/a	n/a	n/a	0.61	n/a	7.72
**H390**	H390D	missense variant	1168C>G (p.His390Asp)	OCA	**Pathogenic**	0.29	0.43	3.99
	n/a	synonymous variant	1170T>C (p.His390=)	not provided	**Likely benign**		n/a	
**P406**	P406L	missense variant	1217C>T (p.Pro406Leu)	OA with congenital sensorineural hearing loss	**Pathogenic/Likely pathogenic**	0.92	0.98	16.35

25 ClinVar-listed mutations mapped to 23 key substrate-interacting residues. The OCA1B-associated P406L mutation is colored red.
*ClinVar Web, **NEI Commons Ocular Proteome Web (https://neicommons.nei.nih.gov/#/proteomeData)

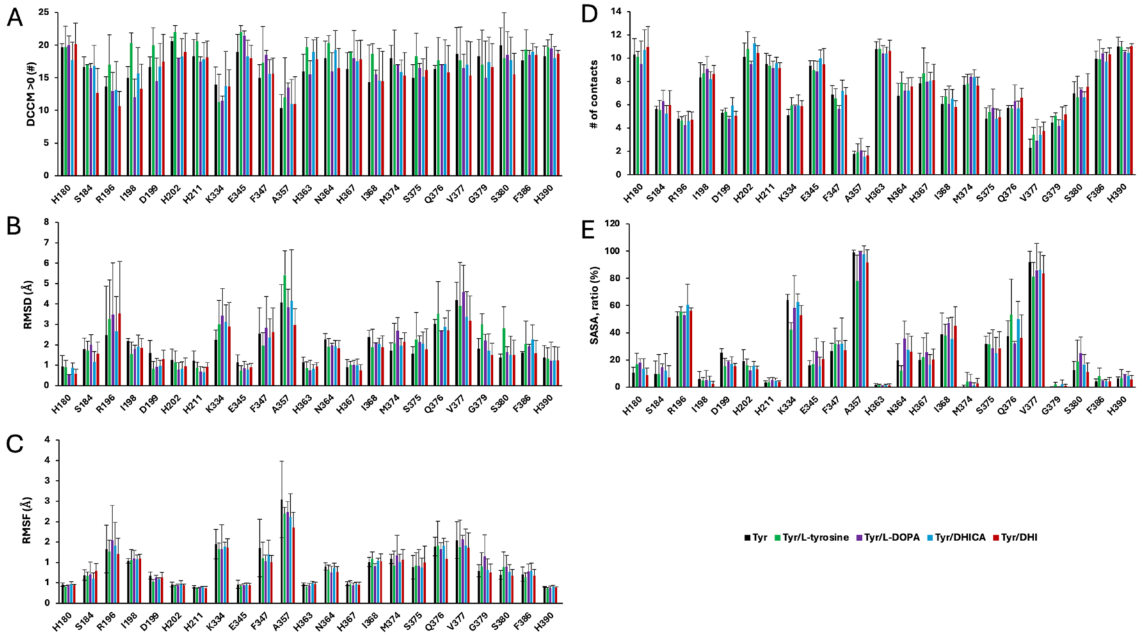

Figure S1. Tyr's substrate-induced structural and dynamic changes. DCCM **(Panel A)**, RMSD **(Panel B),** RMSF **(Panel C),** per-residue number of contacts (**Panel D**), and SASA (**Panel E**) are shown for ligand-interacting residues of Tyr (black bars) and Tyr in complex with L-tyrosine (green bars), L-DOPA (purple bars), DHICA (blue bars), and DHI (red bars). For the ligand-bound condition, values shown on the ordinate axes of Panels A-E represent the averages over the 20 ns MD trajectory obtained for the valid docking poses that remained stable bound to protein structure during MD simulation.

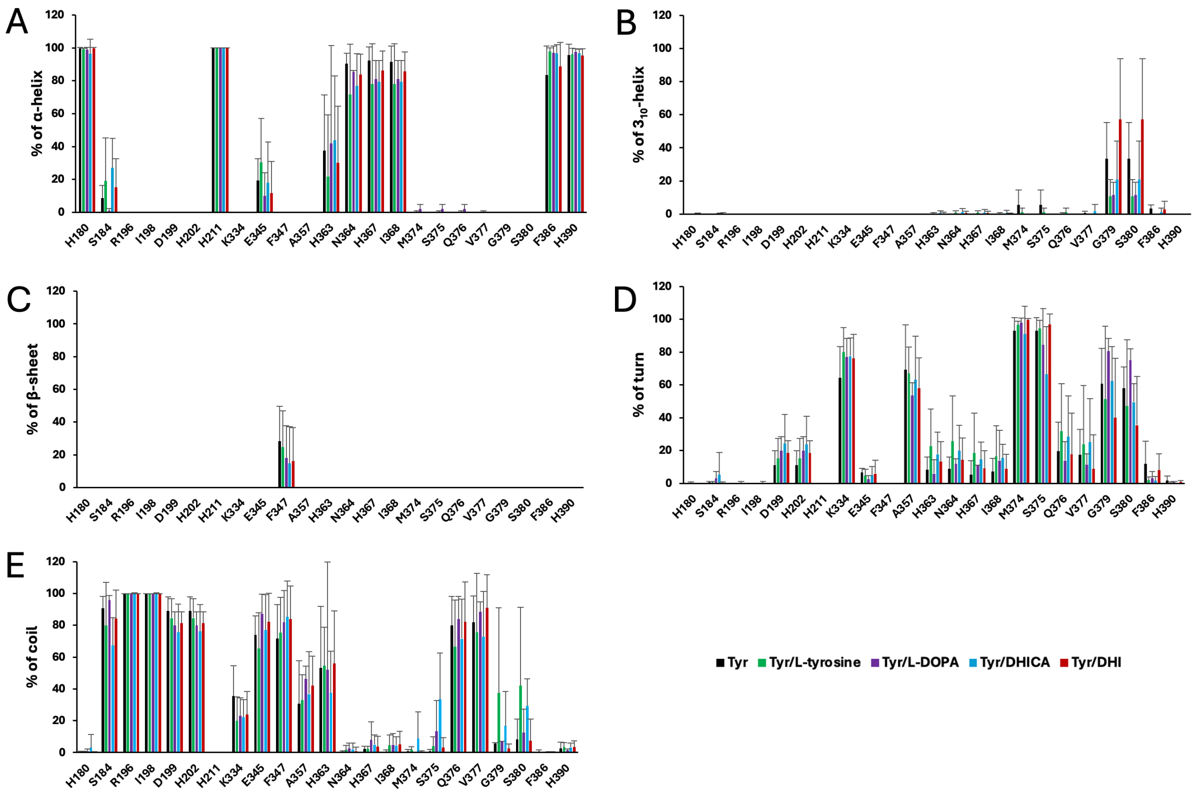

Figure S2. Per-residue secondary structure content of Tyr's ligands-interacting residues. α-helix **(Panel A),** 3_{10}-helix **(Panel B),** β-sheet **(Panel C),** turn **(Panel D),** and coil **(Panel E)** are shown for unbound Tyr (black bars) and Tyr in complex with L-tyrosine (green bars), L-DOPA (purple bars), DHICA (blue bars), and DHI (red bars). For the ligand-bound condition, values shown on the ordinate axis of Panels A-E represent the averages over the 20 ns MD trajectory obtained for the valid docking poses that remained stable bound to protein structure during MD simulation.

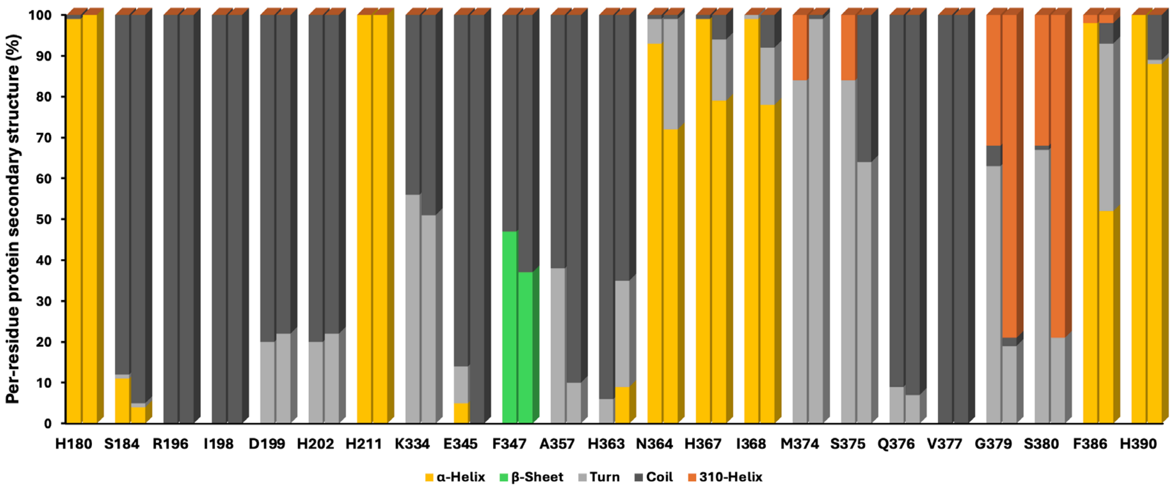

Figure S3. Per-residue secondary structure changes upon the P406L mutation, predicted for Tyr's ligand-interacting residues. Bars represent the secondary structure content for each residue in Tyr (first bars) and the P406L mutant variant (second bars). Secondary structure types are color-coded as follows: α -helix (yellow), β-sheet (green), turn (light grey), coil (dark grey), and 3_{10}-helix (orange).

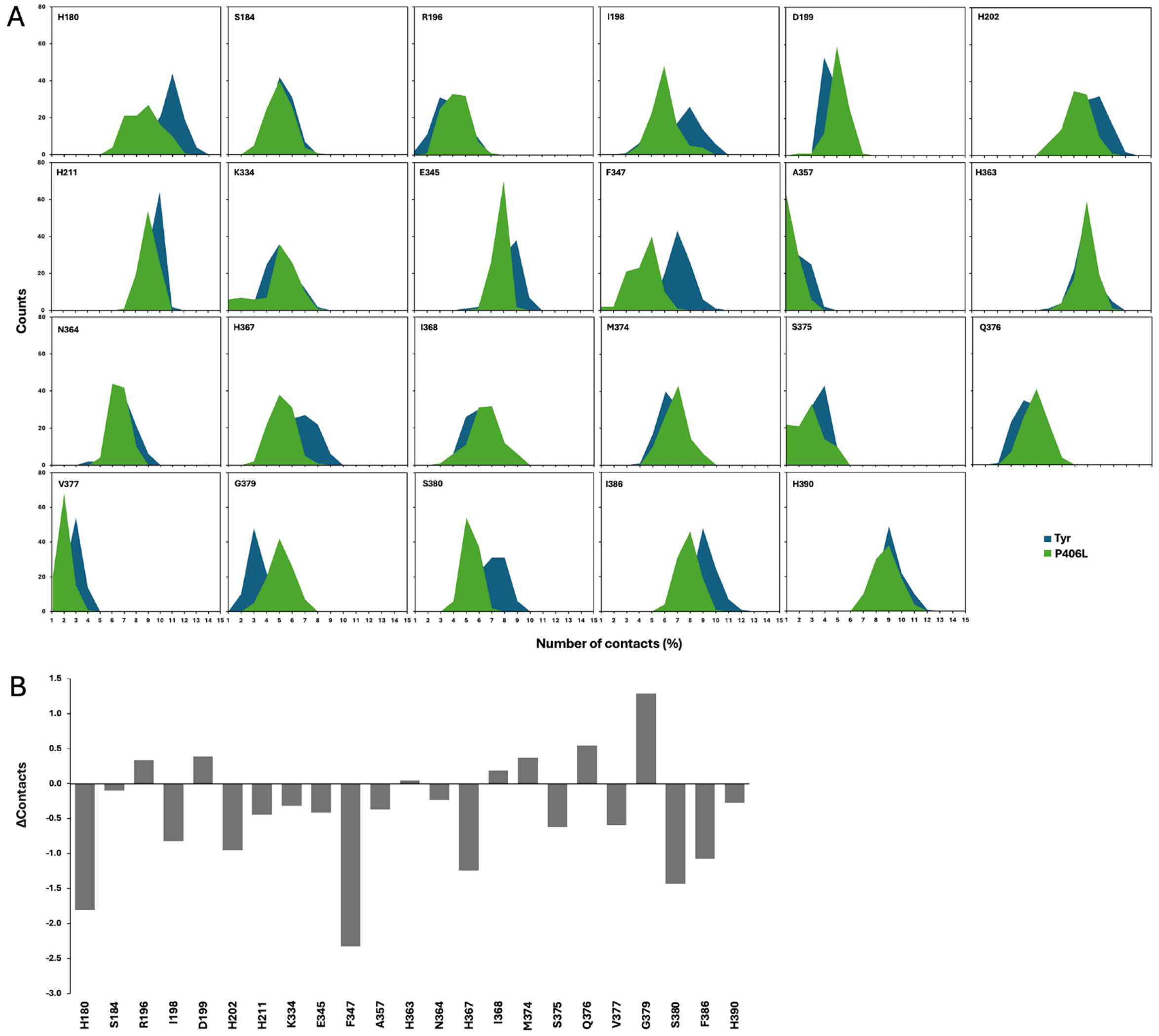

Figure S4. Contact changes in ligand-interacting residues of the P406L mutant variant. Panel A: Per-residue number of contacts for ligand-interacting residues in Tyr (blue), and in the P406L mutant variant (green). **Panel B:** ΔContacts = (Contacts$_{P406L}$ − Contacts$_{Tyr}$) for ligand-interacting residues, highlighting changes induced by the P406L mutation.

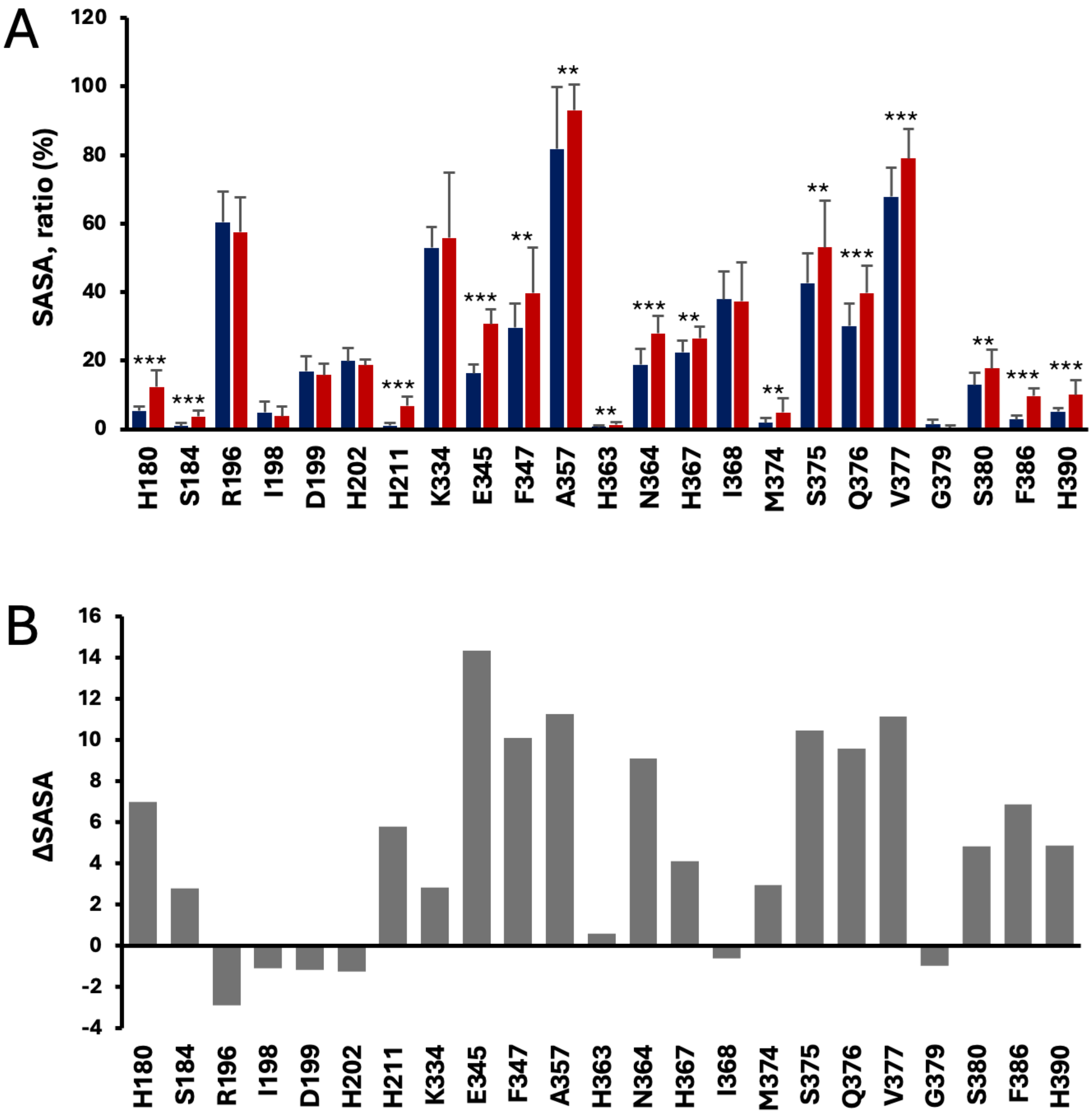

Figure S5. Solvent accessibility changes in ligand-interacting residues of the P406L mutant variant. Panel A: Averaged SASA values (%) with standard deviation over 20 ns of MD simulation for Tyr's ligand-interacting residues in Tyr (blue bars) and P406L (red bars). Statistical significance is indicated as follows: $p<0.05$ (*), $p<0.01$ (**), and $p<0.001$ (***). **Panel B:** $\Delta SASA = (SASA_{P406L} - SASA_{Tyr})$ for ligand-interacting residues, highlighting per-residue changes in solvent exposure upon the P406L mutation.

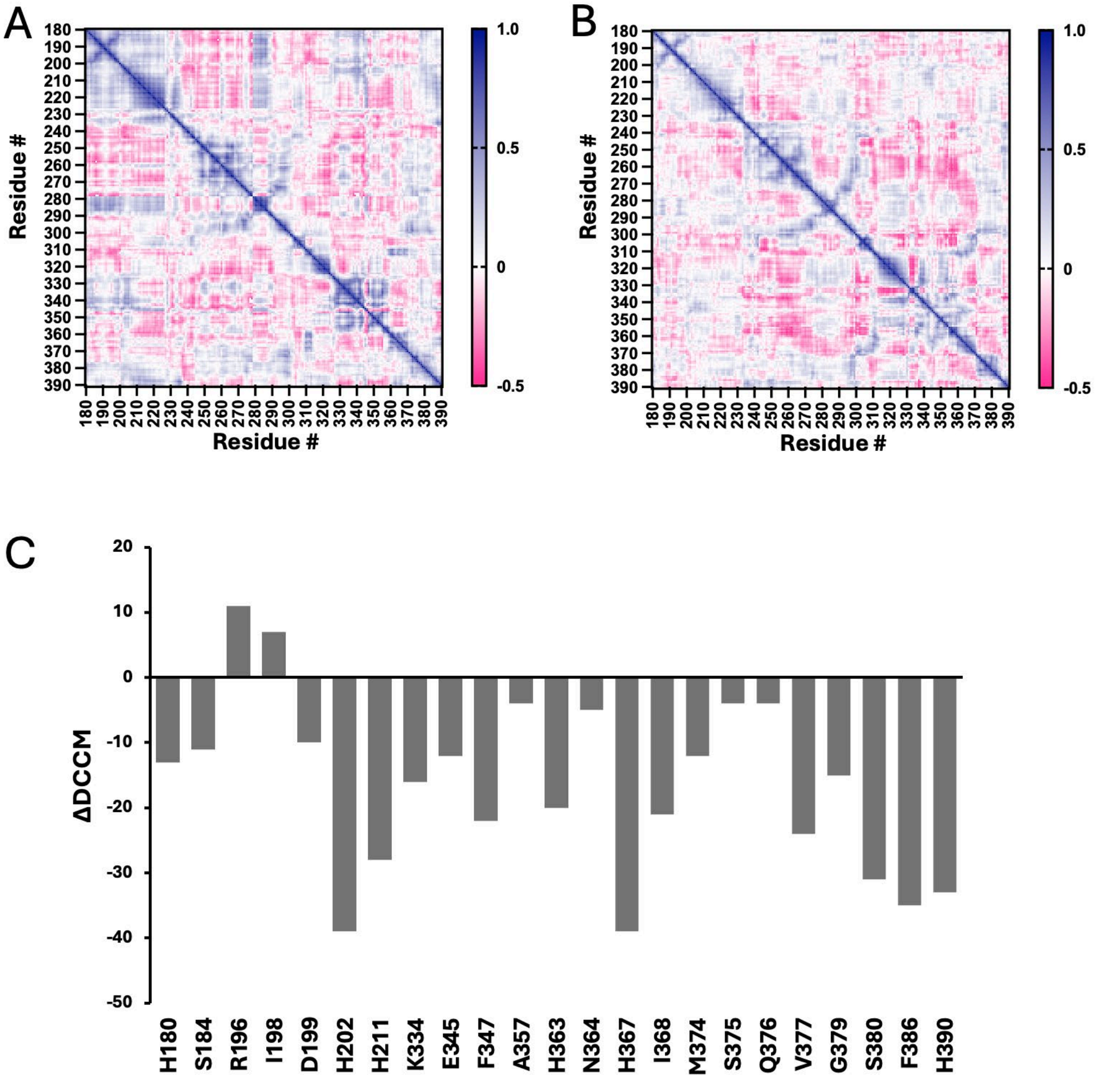

Figure S6. Dynamic correlation changes in the P406L mutant variant of Tyr. DCCM plots of residues 180–390 for Tyr (**A**) and the P406L mutant variant (**B**), highlighting differences in dynamic correlations. The scale bar indicates positive (blue) and negative (pink) correlations. **Panel C:** ΔDCCM = ($DCCM_{P406L}$ – $DCCM_{Tyr}$) for ligand-interacting residues, showing mutation-induced changes in correlated motions.

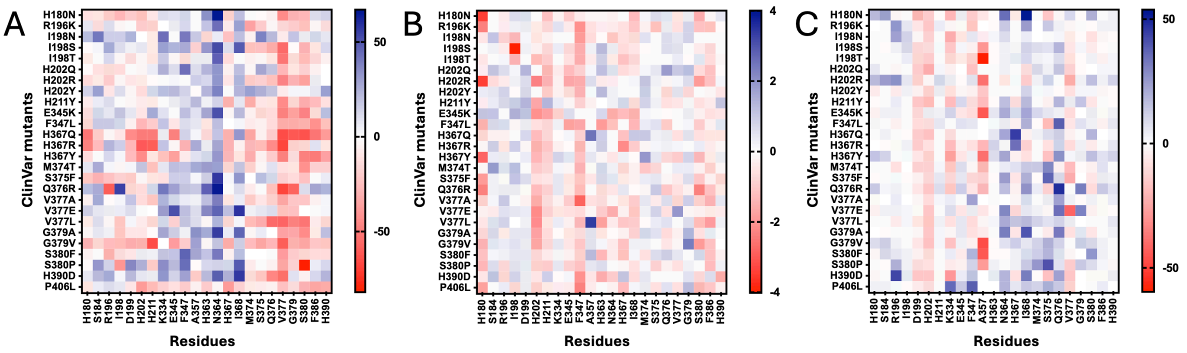

Figure S7. Effect of ClinVar-listed mutations on Tyr active site dynamics. Panel A: ΔDCCM of Tyr's ligands-interacting residues affected by ClinVar-listed mutations. The scale bar represents positive (blue) and negative (red) correlations. **Panel B:** Changes in per-residue contacts of Tyr's ligands-interacting residues affected by ClinVar-listed mutations. The scale bar indicates increases (blue) and decreases (red). **Panel C:** Changes in solvent accessibility of Tyr's ligands-interacting residues affected by ClinVar-listed mutations. The scale bar indicates larger values (blue) and smaller values (red).

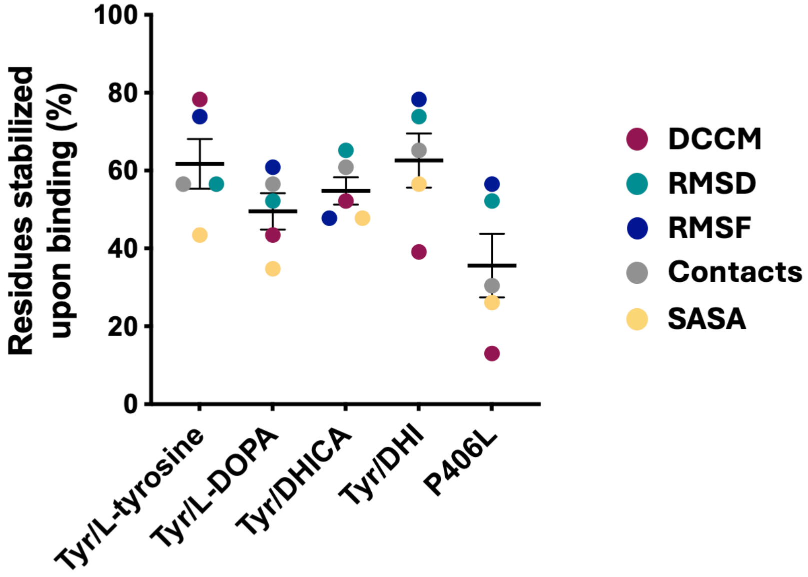

Figure S8. Substrate-dependent stabilization of Tyr and its destabilization by P406L mutation.

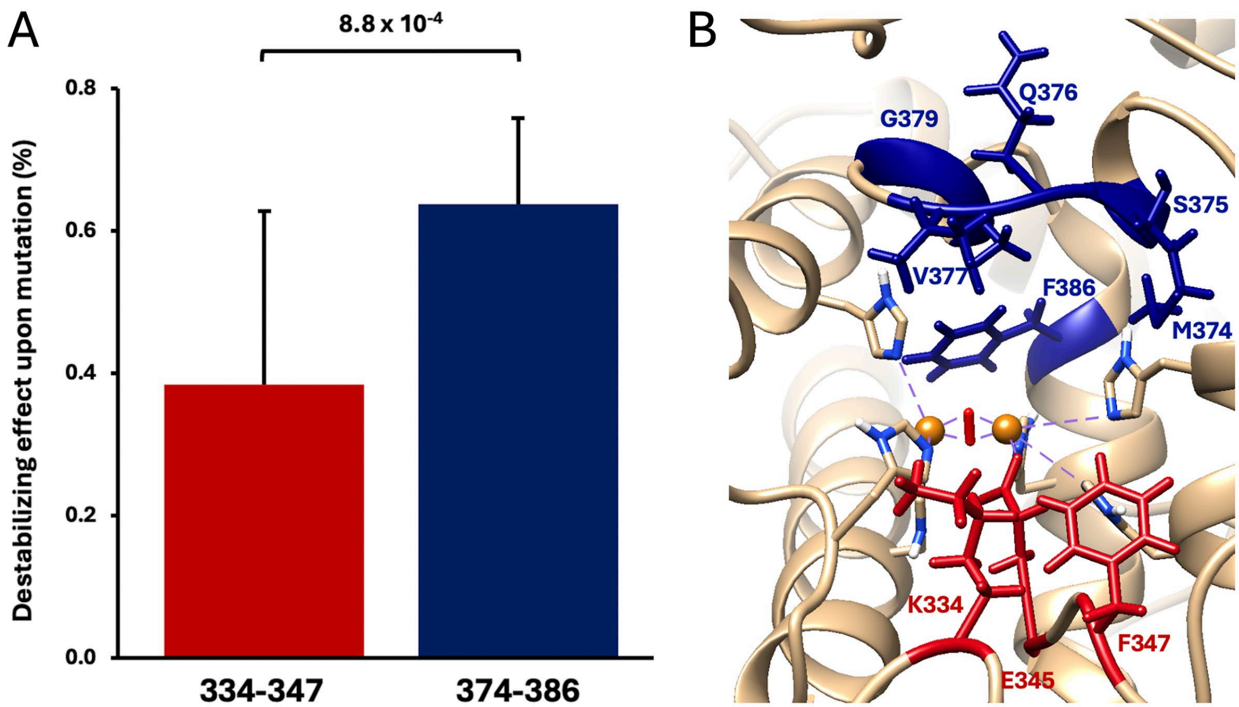

Figure S9. Destabilizing effects of mutations within residues 334–347 and 374–386. Panel A: The anchoring region (residues 334-347) and the flexible gating loop (residues 374-386) are shown as red and blue columns, respectively. Bars represent the average group-level disruption scores calculated from mutation-induced changes in structural and dynamic parameters (RMSD, RMSF, SASA, intramolecular contacts, and DCCM) across analyzed ClinVar variants. Statistical significance was assessed using a two-tailed Student's t-test ($t = 2.2$, $p = 0.00088$). **Panel B:** Homology model of the Tyr intra-melanosomal domain showing residues 334-347 and 374-386 more (blue) or less (red) destabilized upon ClinVar-listed mutations. The protein backbone is shown as a tan ribbon, and copper atoms in the active site are represented as orange spheres.
